# TiO_2_ Nanotubes with Pt and Pd Nanoparticles as Catalysts for Electro-Oxidation of Formic Acid

**DOI:** 10.3390/ma13051195

**Published:** 2020-03-06

**Authors:** Marcin Pisarek, Piotr Kędzierzawski, Mariusz Andrzejczuk, Marcin Hołdyński, Anna Mikołajczuk-Zychora, Andrzej Borodziński, Maria Janik-Czachor

**Affiliations:** 1 Institute of Physical Chemistry, Polish Academy of Sciences, Kasprzaka Str. 44/52, 01-224 Warsaw, Poland; pkedzierzawski@ichf.edu.pl (P.K.); mholdynski@ichf.edu.pl (M.H.); amikolajczuk@ichf.edu.pl (A.M.-Z.); aborodzinski@ichf.edu.pl (A.B.); mczachor@ichf.edu.pl (M.J.-C.); 2 Faculty of Materials Science and Engineering, Warsaw University of Technology, Woloska Str. 141, 02-507 Warsaw, Poland; mariusz.andrzejczuk@pw.edu.pl

**Keywords:** TiO_2_ nanotubes, Pt and Pd nanoparticles, surface and structure characterization, electrocatalysis, formic acid fuel cell

## Abstract

In the present work, the magnetron sputtering technique was used to prepare new catalysts of formic acid electrooxidation based on TiO_2_ nanotubes decorated with Pt (platinum), Pd (palladium) or Pd + Pt nanoparticles. TiO_2_ nanotubes (TiO_2_ NTs) with strictly defined geometry were produced by anodization of Ti foil and Ti mesh in a mixture of glycerol and water with ammonium fluoride electrolyte. The above mentioned catalytically active metal nanoparticles (NPs) were located mainly on the top of the TiO_2_ NTs, forming ‘rings’ and agglomerates. A part of metal nanoparticles decorated also TiO_2_ NTs walls, thus providing sufficient electronic conductivity for electron transportation between the metal nanoparticle rings and Ti current collector. The electrocatalytic activity of the TiO_2_ NTs/Ti foil, decorated by Pt, Pd and/or Pd + Pt NPs was investigated by cyclic voltammetry (CV) and new Pd/TiO_2_ NTs/Ti mesh catalyst was additionally tested in a direct formic acid fuel cell (DFAFC). The results so obtained were compared with commercial catalyst—Pd/Vulcan. CV tests have shown for carbon supported catalysts, that the activity of TiO_2_ NTs decorated with Pd was considerably higher than that one decorated with Pt. Moreover, for TiO_2_ NTs supported Pd catalyst specific activity (per mg of metal) was higher than that for well dispersed carbon supported commercial catalyst. The tests at DFAFC have revealed also that the maximum of specific power for 0.2 Pd/TiO_2_ catalyst was 70% higher than that of the commercial one, Pd/Vulcan. Morphological features, and/or peculiarities, as well as surface composition of the resulting catalysts have been studied by scanning electron microscopy (SEM), scanning transmission electron microscopy (STEM), and chemical surface analytical methods (X-ray photoelectron spectroscopy—XPS; Auger electron spectroscopy—AES).

## 1. Introduction

The development of nanotechnology has brought many opportunities in the design of functional materials by electrochemical methods, leading to, for example, preparation of oxide nanostructures on metals/alloys in the form of freestanding nanotubes or nanoporous layers. Since the time when the first papers on the fabrication of titania nanotubes on titanium by anodic oxidation [1,2] appeared, determining their growth mechanism and physicochemical properties [3], there has been a continuous attempt to find potential applications for such materials [4]. Titanium oxide nanotubes (TiO_2_ NTs) have become the subject of intensive research because of their specific morphology, which is relatively easy to modify by changing the parameters of titanium anodic oxidation like electrolyte, voltage and anodization time. These changes leads to direct linear relation between applied voltage and the average diameter of the nanotubes formed. Thus, the diameter of TiO_2_ NTs usually increases with the anodic voltage [3,4,5,6]. Formation of TiO_2_ NTs with various diameter, length, shape and wall thickness leads to control the tubes geometric surface area (specific surface area). These parameters are crucial for preparation of the materials which may find applications in electrocatalysis/energy production [7,8,9], heterogeneous catalysis [4,10], and photovoltaics [4,11]. Apart from the geometric advantages of nanoporous layers, whose growth is perpendicular to the metal substrate, they feature a uniform chemical composition and structure that can be controlled by suitable thermal treatment (at temperatures above 400 °C). The annealing temperature, when properly chosen, makes it possible to convert the structure of the nanotubes from amorphous (directly after anodic oxidation) to crystalline: anatase, rutile, and/or a mixture thereof [5,6,12,13,14]. Therefore, the vertically oriented tubular structures (with a specific crystal structure) feature a high degree of electron mobility along the tube axis perpendicular to the titanium substrate [15]. Other important factors are the properties of this material, which are the result of the nature of titanium oxide as: corrosion resistance over a wide range of pH, good catalytic properties due to surface formation of reactive oxygen species (ROS), no harm to the environment, relatively inexpensive material [16,17,18,19]. All these properties are interesting from the point of view of TiO_2_ NTs applications in elctrocatalysis [7,8,9]. Thus, in comparison with carbon nanostructures commonly used in this kind of applications, the TiO_2_ NTs with deposited noble metals may show good electrocatalytic performance, taking into account the advantages mentioned above and superior connectivity between metal nanoparticles and oxide support [20]. The nanoporous oxide support can change electronic properties of the metal by so called strong metal-support interaction (SMSI). This is due to the effect of electron transfer from TiO_2_ lattice to metal nanoparticles [21]. The problem that may arise with nanotubes is that the semi-conductive oxides have a poor electrical conductivity. To the best of our knowledge, there are only a few papers in the literature where TiO_2_ nanotubes were applied as a catalyst support—e.g., for electrooxidation of formic acid [7,8]. Therefore, it is crucial to understand how the structural, morphological and physicochemical properties of functionalized TiO_2_ nanotubes could affect fuel cell performance. Designing new liquid fuel cells based on new catalysts requires their optimization, but some works are also focused on developing new methods for the functionalization of currently used catalysts [8]. The TiO_2_/C composites have already been employed as an anodic catalyst support in a DFAFC [22]. Fuel cells convert directly chemical energy to electrical one with high efficiency not limited by the Carnot principle. The most advanced low temperature fuel cell uses hydrogen as a fuel, however, because of hydrogen storage problems, there is intensive research on fuel cells using liquid fuels. At the moment, DFAFC is the most promising among them. The important advantage of FA (formic acid) as a fuel in contrast to fossil fuels is the possibility of FA production from biomass or sequestrated CO_2_, which will reduce CO_2_ emission to the environment. Moreover, formic acid has a number of the advantages over methanol as a fuel: faster oxidation reaction, high theoretical thermodynamic cell potential, and lower fuel crossover through the ion conducting membrane. In contrast to methanol, formic acid is a nontoxic liquid and dilute formic acid solutions are safe: they are used as a food additive. When released to environment it decomposes easily [23,24,25].

Pt is a commonly used metal in this type of electrochemical reaction and is frequently used as a reference material for systems which contain other catalytically active metals, such as Pd [26]. Partial substitution of Pt with some other metal—e.g., Au or Pd—might also be an effective way to increase the catalytic activity in the formic acid oxidation reaction. It has been known since 2005 that the electrocatalytic activity of Pd for formic acid oxidation is very high [27]. The drawback is that to maintain good performance and stability it requires formic acid of high purity (HPLC grade—Ultra High Purity) what drives high running costs of the fuel cell. Use of an inexpensive, technical grade formic acid causes anode catalyst poisoning and quick drop in the performance [28,29]. On the other hand, Pt is readily poisoned by carbon monoxide, a reaction intermediate. One of the ways of removing the poison is to introduce oxidizing centers, which oxidize CO adsorbed on platinum to CO_2_. The centers can come from the alloying element such as Ru (ruthenium) or Ir (iridium) [30], decoration with another element [31], but also from the catalyst support [7]. Furthermore, it is often observed that the carbon supports during the reaction undergo severe corrosion which leads to the decrease of the electrochemically active surface area of the catalyst due to the detachment of the metal nanoparticles (e.g., Pt) from the support [18,19]. Thus, designing new catalysts for fuel cells based on a TiO_2_ nanoporous oxide layer is a promising alternative to the commonly used substrates based on various carbon structures [21,32,33]. The TiO_2_ NTs have been used in the reaction of methanol electro-oxidation [8], and the evolution of hydrogen [22].

In the present work, the magnetron sputtering technique was used to prepare new catalysts supported on TiO_2_ nanotubes with Pt, Pd, or Pd + Pt nanoparticles. The catalytic activity is strongly dependent on the shape, size, and distribution of the metal particles. Therefore, adequate methods are required that could control these important factors. Magnetron sputtering appeared a useful technique for catalysts preparation, because it allows a precise control over the thickness, composition and structure of the deposits. The important advantage of this method of metal deposition over most frequently used chemical methods is that it does not introduce any chemical impurities to the catalyst. Therefore, it can be used for preparation of well-defined multi-metallic nanoparticles. The structures thus obtained were then tested as anode materials: (1) for formic acid electrooxidation in 0.5 M H_2_SO_4_ and also (2) for working anode in a DFAFC.

## 2. Experimental Methods

### 2.1. Sample Preparation

TiO_2_ NT nanoporous layers were fabricated by anodic oxidation of Ti samples: (i) for CV measurements—Ti foil, 0.25 mm-thick, 99.5% purity 99.5%, Alfa Aesar, (Haverhill, MA, USA) (ii) for fuel cell tests—Ti mesh, 135 MESH, smooth weave type, 0.058 mm wire thickness. An optimized electrolyte based on glycerol (52 wt %) and deionized (DI) water (47.14 wt %) + with ammonium fluoride (0.86 wt %), a constant voltage of 25 V and 40 min time of anodization were employed. The anodic titanium oxidation process was carried out in a two-electrode system, where the Ti foil and mesh was an anode (+) and the Pt mesh (in the form of square or cylinder) was a cathode (−). Using these conditions, the growth of TiO_2_ nanotubes is perpendicular to the metal substrate [6]. The diameter and length of the nanotubes is about 100 nm and 800 nm, respectively [6]. After anodic oxidation treatment, the all samples were cleaned with DI water for 24 h and dried in air. In the next step, the heat treatment in air was performed at 450 °C for 1 h. This treatment leads to transformation of TiO_2_ NTs structure from amorphous to crystalline phase: anatase.

### 2.2. Deposition of Metal Nanoparticles

The fabricated titania nanostructures after their annealing process were covered with Pt (0.2 mg cm^−2^, one cycle), Pd (0.2 mg cm^−2^, one cycle) or Pd (0.1 mg cm^−2^, one cycle) + Pt (0.02 mg cm^−2^, second cycle) by the DC magnetron sputtering technique using a Leica EM MED020 apparatus (Leica Mikrosystems GmbH, Wetzlar, Germany). For bi-metallic sample a small amount of Pt was deposited on the top of Pd layer to decorate the Pd metallic surface and create catalytically active sites as a junction of Pd and Pt. The purity of the targets used was above 99.9%. Before sputtering, a vacuum chamber was pumped down to a pressure of 3 × 10^−3^ Pa and rinsed several times with high purity Ar (argon, 99.99%). The glow plasma discharge was obtained in an argon atmosphere at a pressure of 2.0 Pa and 25 mA current. Before deposition, the target was pre-sputtered for about 30 s to remove contaminants from the target surface. The average amount of metal deposited per cm^2^ was strictly controlled by quartz microbalance (Leica EM QSG100—Leica Microsystems GmbH, Wetzlar, Germany) in situ measurements. The configuration of the setup was perpendicular to the surface of the sample.

### 2.3. Membrane Electrode Assemblies (MEAs) Preparation

The Pd/TiO_2_ nanotubes/Ti mesh was prepared as follows: Ti mesh (135 MESH, smooth weave type, 0.058 mm wire thickness) was anodized in the same way as Ti foils to produce TiO_2_ nanotubes. Pd nanoparticles were subsequently deposited by sputtering on two sides of the TiO_2_ nanotubes/Ti mesh substrate as described for the foil. Before MEA (membrane electrode assemblies) pressing, the Ti mesh with the catalyst was covered with 5% Nafion^®^ suspension (DE520, Du Pont, Wilmington, DE, USA) dissolved in ethanol. The other electrodes were prepared by covering carbon fabrics with an ink of catalyst and a dispersion of ionic conductor. For the anode used as reference 20% Pd/Vulcan from Premetek (Premetek, Cherry Hill, NJ, USA) was employed. For cathodes 60% Pt/Vulcan from Premetek was used as a catalyst. Catalyst inks were prepared by sonication of catalysts particles with water and 5 wt % Nafion^®^ (Wilmington, DE, USA) emulsion (DE520, Du Pont) and sonicated for 30 min. The ink was dispersed on a carbon cloth (anode: B1B from BASF Fuel Cells (Ludwigshafen, Germany), no wet proofing, cathode: B1B30WP from BASF Fuel Cells, 30 wt % wet proofing). Pd loading of the catalyst on the anode was 0.5 mg cm^−2^ and Pt loading on the cathode was 4.0 mg cm^−2^. The 5 cm^2^ MEAs were prepared by hot pressing (for 10 min at 130 °C and 0.8 kg cm^−2^) of Nafion^®^ 115 membrane, with an anode and a cathode.

### 2.4. SEM

For the visualization of the morphology of the received samples after each step of preparation, examinations were carried out with a scanning electron microscope FEI NovaNanoSEM 450 (FEI Company, ThermoFisher Scientific, Brno, Czech Republic). For this purpose, secondary electron detector (SE-TLD) and accelerating voltage 5 or 10 kV were applied.

### 2.5. STEM

Microstructure and structural investigations were performed using a Hitachi HD-2700 high resolution scanning transmission electron microscope -HR-STEM (Naka, Japan) operating at 200 kV and equipped with X-ray energy dispersive spectroscopy system -EDS (Thermo Scientific, Waltham, MA, USA) for chemical analysis. The following STEM modes were used for acquisition of high resolution images: bright field (BF) and high-angle annular dark-field (HAADF), which provides Z-contrast. TEM examinations were performed on thin samples prepared by a Hitachi NB5000 (Naka, Japan) focused ion beam (FIB) system. The cross-sectional samples after FIB method (lamellas) were additionally thinned using low-energy argon ion milling on a Gentle Mill (Technoorg Linda Ltd., Budapest, Hungary). Samples prepared in this way were used for STEM microscopic observations.

### 2.6. XPS/AES

The chemical composition and chemical state of the oxide layers before and after Pt, Pd, and Pd + Pt deposition processes by magnetron sputtering were examined using photoelectron spectroscopy (XPS) and Auger electron spectroscopy (AES). For this purpose the Microlab 350 (VG Thermo Scientific, East Grinstead, UK) was used. The high-resolution XPS spectra were recorded using 40 eV pass energy with the 0.1 eV step at the excited energy 1486.6 eV (AlKα). A Smart function (modified Shirley function) was used to cut-off the background to obtain the XPS signal intensity for individual elements. Next, the all measured peaks were deconvoluted using an asymmetric Gaussian/Lorentzian mixed function. The determined binding energies in this case were corrected to the energy of C 1s peak at 284.5 eV, as reference BE position. Furthermore, the local Auger spectra were recorded at excited energy—10 kV. Avantage software (Version 5.9911, ThermoFisher Scientific, Waltham, MA, USA) was used for XPS and AES data processing.

### 2.7. CV

Electrochemical measurements were performed in the standard three-electrode electrochemical cell with a Pt gauge as counter electrode and Ag/AgCl│1 M KCl as reference electrode. The electrolyte solution (0.5 M H_2_SO_4_ or 0.5 M H_2_SO_4_ + 0.5 M HCOOH) was purged before the measurements with high purity N_2_. CV curves were recorded at ambient temperature (22 ± 1 °C) using an EP-20 potentiostat with an EG-20 function generator from Elpan (Lubawa, Poland). Electrochemically available surface areas (EAS) of deposited metals onto TiO_2_ nanotubes were measured in 0.5 M H_2_SO_4_. Reference commercial 20% Pd/Vulcan from Premetek was deposited on glassy carbon disk in the form of ink. 2 mg of catalyst and 25 μL of 5% Nafion dispersion were dissolved in 2 mL of ethanol and ultrasonicatated for 30 min. 0.5 mL of the ink was deposited on the 1 cm^2^ glassy carbon working electrode and then dried at 80 °C. Formic acid electrooxidation current densities are normalized per total mass of metals.

### 2.8. Fuel Cell Tests

The DFAFC (direct formic acid fuel cells) tests were performed by measuring polarization curves. Current-voltage characteristics of the fuel cell were measured using a home built voltage controlled galvanostat and recorded with a data acquisition system (software, version 1.0, Institute of Physical Chemistry PAS, Warsaw, Poland). MEAs were placed between current collectors made of graphite with serpentine flow channels. 3 M aqueous formic acid for HPLC (09676 Fluka—Ultra High Purity) solution was supplied on the anode at the flow rate of 2.1 mL min^−1^. On the cathode, oxygen at the flow rate of 1.0 L min^−1^ was employed. Fuel cell tests were carried out at the temperature of 30 °C.

## 3. Results and Discussions

### 3.1. Characterization of the Catalysts

**Figure 1:**
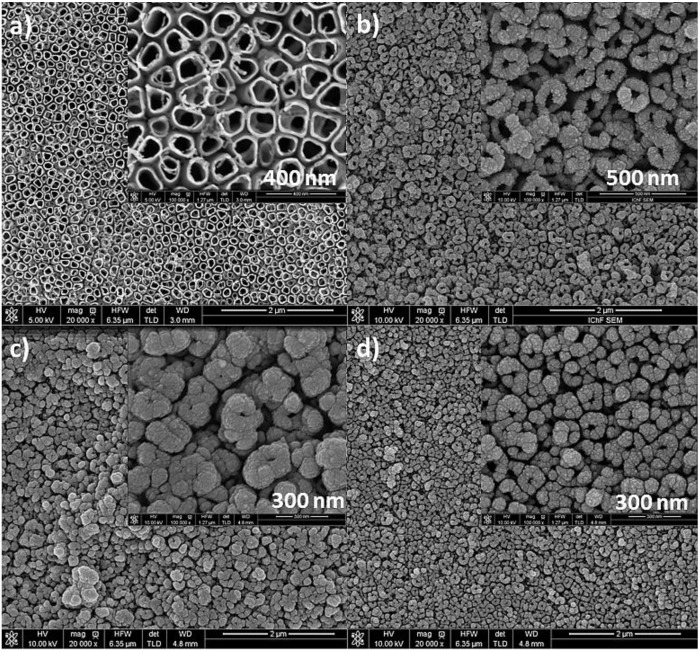
SEM images of a nanotubes formed at 25 V, annealed at 450 °C/1h (**a**) with a metal deposits: 0.2 mg cm^−2^ Pt (**b**), 0.2 mg cm^−2^ Pd (**c**), 0.1 mg cm^−2^ Pd + 0.02 mg cm^−2^ Pt (**d**).

Figure 1 shows SEM images of the surface morphology of titanium oxide nanotubes fabricated at 25 V, annealed at 450 °C for 1 h in air (a) with 0.2 mg cm^−2^ Pt (b), 0.2 mg cm^−2^ Pd (c) and 0.1 mg cm^−2^ Pd + 0.02 mg cm^−2^ Pt (d) deposits. The nanotubes before magnetron sputtering process are hollow in shape and separated from each other (Figure 1a). After deposition process metal nanoparticles tend to occupy the edges of the TiO_2_ nanotubes with upper part of their side walls still reflecting their original morphology. Rings consisting of agglomerates of Pt or Pd nanoparticles are well visible in Figure 1b–d. Moreover, the size of nanoparticles deposited on the surface of nanotubes varies depending on the type of metal used. This is due to the different atomic mass, density, and melting point of both metals. Under magnetron sputtering conditions, Pt tends to form smaller nanoparticles than Pd.

**Figure 2:**
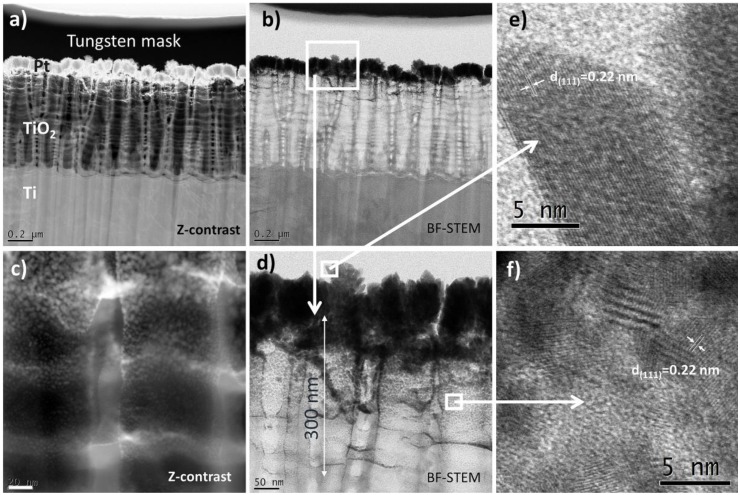
Cross-sectional view of titania nanotubes (25 V, 450 °C/1 h) decorated with 0.2 mg cm^−2^ Pt. STEM images obtained via Z-contrast (**a**,**c**) and BF-contrast (**b**,**d**), as indicated. (**e**) and (**f**) shows HR STEM images of the single nanotube and Pt nanoparticles attached to the walls of tubes.

More detailed information about morphology and distribution of the deposited metal nanoparticles provide STEM investigations. It is evident that the diameter of the nanoparticles changes with the height of the nanotubes—from the top to the bottom. At the nanotube tops there are large agglomerates of Pt particles, which maps the nanoporous substrate: Figure 2a,b. The tendency to such an agglomeration diminishes with nanotube depth. In deeper locations within the nanotubes, small Pt particles of 2–5 nm in diameter are clearly visible (see Figure 2c). The high resolution investigations revealed also that the Pt nanoparticles may occupy locations at the nanotube walls even at a depth of 300 nm from the top of the oxide layer (see Figure 2d). All the particles, both those in the agglomerates and these separated one from another, are crystalline (see Figure 2e,f). The HR-STEM images suggest that the interplanar distance between neighboring crystallographic planes is about 0.22 nm, and corresponds to the Pt (111) crystal plane.

**Figure 3:**
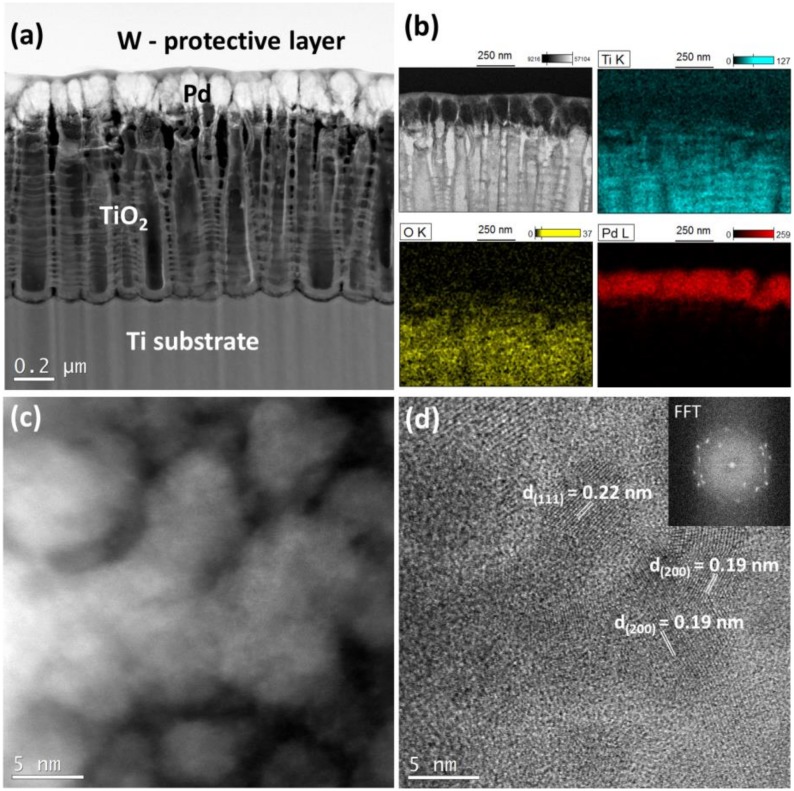
Cross-sectional view of titania nanotubes (25 V) decorated with Pd (magnetron sputtering technique) (**a**) HAADF-STEM image with visible layers, (**b**) EDS elemental maps indicating presence of Pd, (**c**) HAADF-STEM image taken from the Pd layer, and (**d**) high resolution STEM image showing a lattice spacing of Pd (111) and (200).

In the case of palladium, a similar arrangement was found on the surface of the TiO_2_ nanotubes. The metal covers the walls of certain nanotubes forming thin solid coatings around them (Figure 3a). EDS maps, Figure 3b, confirm the existence of Pd particles deep inside the NTs layer. However the majority of Pd mass is located on top of the TiO_2_ NTs. Some small metal particles of the size below 10 nm are also visible on the walls of TiO_2_ NTs (Figure 3c). The crystallinity of the deposited Pd nanoparticles is well visible in Figure 3d. The lattice spacing was measured as being about 0.22 and 0.19 nm which corresponds to the Pd (111) or (200) crystal planes.

**Figure 4:**
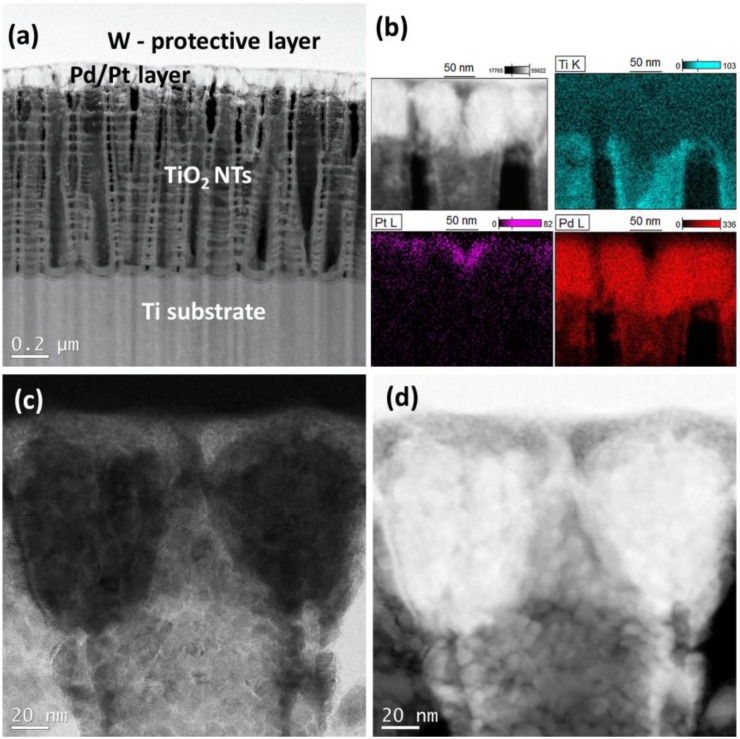
Cross-sectional view of titania nanotubes (25 V) adorned with Pd/Pt (magnetron sputtering technique) (**a**) HAADF-STEM image with visible layers, (**b**) EDS elemental maps indicating presence of Pt and Pd, (**c**) BF-STEM image, and (**d**) HAADF-STEM image of deposited Pd + Pt layer.

A typical cross-sectional view of a bimetallic system Pd + Pt used as an electrocatalyst for formic acid oxidation is shown on Figure 4. It clearly indicates that Pd nanoparticles penetrate the interior of the TiO_2_ nanotubes and the spaces between individual tubes (Figure 4a–d). Deposition of a small amount of Pt (0.02 mg cm^−2^) on the top of Pd coating (0.1 mg cm^−2^) leads to the formation of a specific bimetallic layer, which is shown on Figure 4b. EDS results have shown that the top surface of Pd coating is enriched in Pt, forming a very thin (~20 nm) overlayer with some amount of Pt particles penetrating Pd metal layer. Such a morphology is a result of our procedure applied, where the metals were deposited sequentially: first palladium and then platinum.

**Figure 5:**
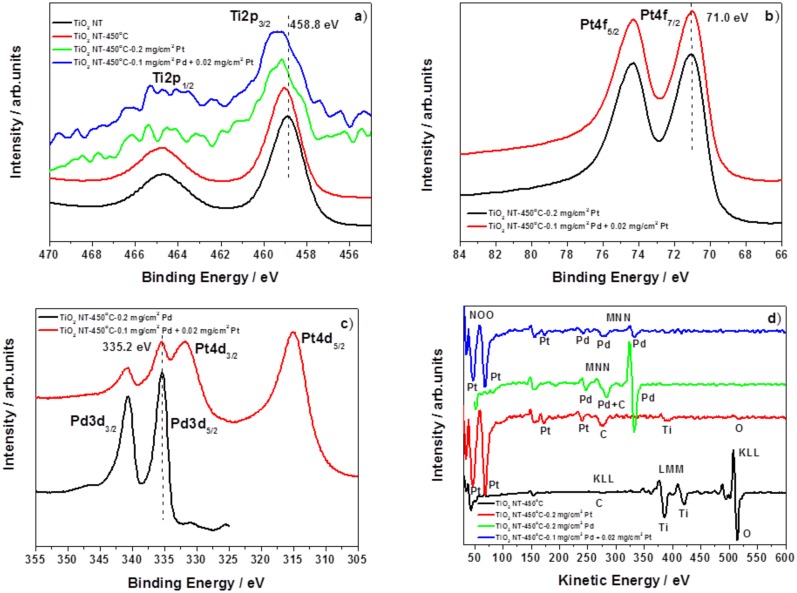
High resolution XPS spectra of Ti2p (**a**), Pt4f (**b**), Pd3d (**c**) regions and AES survey spectra (**d**) registered on the surface: TiO_2_ NT 25 V (as-received), TiO_2_ NT 25 V/450 °C −1h, TiO_2_ NT 25 V/450 °C −1h + 0.2 mg cm^−2^ Pt, TiO_2_ NT 25 V/450 °C −1h + 0.2 mg c^2–2^ Pd, TiO_2_ NT 25 V/450 °C −1h + 0.1 mg cm^−2^ Pd + 0.02 mg cm^−2^ Pt.

XPS (X-ray photoelectron spectroscopy) and AES (Auger electron spectroscopy) results provide an interesting information about the surface composition of the nanostructures prepared. Figure 5 shows XPS high resolution spectra of Ti2p, Pt4f, Pd3d, and AES survey spectra for all investigated samples before and after metal deposition onto TiO_2_ NT support. Figure 5a demonstrates the corresponding Ti2p spectra, these results confirm that TiO_2_ is the main component of the nanotubes subsequently annealed at 450 °C (catalysts support), where the Ti2p_3/2_ peak is located at 458.8 and 459.0 eV, respectively [34]. The XPS spectra for the samples with Pt (0.2 mg cm^−2^) and Pd-Pt (0.1 mg cm^−2^ + 0.02 mg cm^−2^) nanoparticles revealed that the Ti2p signal is shifted in the direction of higher binding energies ca. 0.3 eV as compared to that of heat treated TiO_2_ NT. The shifts detected may suggest that the XPS signals from TiO_2_ are modified by an interaction with deposited metal nanoparticles. This in turn may induce a shift of the Fermi level in the Pt or Pd deposit due to SMSI (strong metal-support interaction) effect [35], where the electron transfer from TiO_2_ lattice (in particular through local defects) to metal nanoparticles may appear [36]. The binding energy peaks of the Pt4f_7/2_ and Pt4f_5/2_ (Figure 5b) were measured at 71.0 and 74.3 eV, respectively. These measured spectral lines suggest that the Pt nano-particles are associated to metallic state for sample with 0.2 mg cm^−2^ Pt and 0.1 mg cm^−2^ Pd + 0.02 mg cm^−2^ Pt. The deconvolution of this peak for both samples has also revealed presence of Pt oxides on the surface of electrocatalysts investigated: PtO (72.2–72.3 eV) and PtO_2_ (73.4–73.6 eV) [35,37]. The percentages of the metallic state of Pt was calculated basing on the integration of its individual components to the total amount of Pt. It was found that 70% of Pt is in its metallic state.

Figure 5c shows Pd3d region of XPS spectra of the TiO_2_ NT decorated by Pd (0.2 mg cm^−2^) and Pd + Pt (0.1 mg cm^−2^ + 0.02 mg cm^−2^) nanoparticles; characteristic peaks originating from the metal deposit are visible on the spectra. Detailed inspection of XPS spectra suggests the presence of metallic Pd on the surface of the catalyst: Pd3d_5/2_–335.2 and 335.3 eV, respectively [26]. The Pd3d_5/2_ core level spectra contain also two peaks at ~336.0 and ~338.0 eV which may be assigned to Pd in PdO and/or to PdO_2_. These XPS results suggest that the surface of Pd deposit is partly oxidized. Basing on these XPS measurements we may estimate, that ~50% of Pd-containing deposit is in metallic state, for both samples containing palladium. It should be noted, that in the conditions of DFAFC the metal oxides will be rapidly reduced by formic acid [38].

Local Auger spectra shown in Figure 5d confirm qualitatively this finding. The Auger signals of the deposited metallic Pd + Pt containing layers onto TiO_2_ NT support are clearly distinguishable: Pt (NOO), Pd (MNN), Ti (LMM), O (KLL). AES data have revealed that after deposition of Pd nanoparticles (0.2 mg cm^−2^) it was not possible to register signal from the titania support during XPS analysis. Evidently, the metal deposit ‘layer’ is thicker than the information depth for Auger electrons generated from titania support underneath.

### 3.2. Activity of the Electrocatylysts

#### 3.2.1. CV Tests

**Figure 6:**
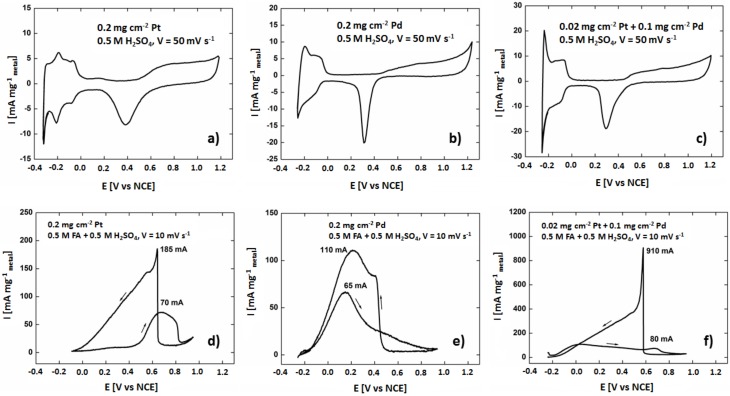
Cyclic voltammograms of the (**a**,**d**) 0.2 mg cm^−2^ Pt/TiO_2_ NT, (**b**,**e**) 0.2 mg cm^−2^ Pd/TiO_2_ NT, (**c**,**f**) 0.1 mg cm^−2^ Pd + 0.02 mg cm^−2^ Pt/TiO_2_ NT catalysts modified electrodes recorded in 0.5 M H_2_SO_4_ and 0.5 M H_2_SO_4_ + 0.5 M HCOOH.

**Figure 7:**
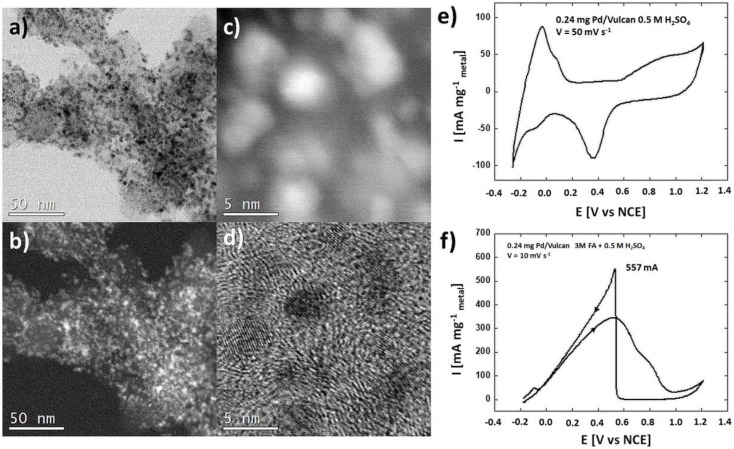
STEM images of the commercial Pd/Vulcan catalyst:(**a**) BF-STEM (Z-contrast), (**b**) HAADF-STEM (Z-contrast), (**c**,**d**) HR STEM images of the single Pd nanoparticles within a carbon matrix, and (**e**,**f**) cyclic voltammograms as measured on this material in 0.5 M H_2_SO_4_ and 0.5 M H_2_SO_4_ + 0.5 M HCOOH (**e**,**f**).

The three different catalysts supported on TiO_2_ nanotubes with 0.2 mg cm^−2^ Pt, 0.2 mg cm^−2^ Pd, and 0.1 mg cm^−2^ Pd + 0.02 mg cm^−2^ Pt deposits were tested in the electro-oxidation of formic acid. Steady state CV curves (after 20 cycles) were registered in 0.5 M sulfuric acid electrolyte and the results are presented in Figure 6a–c. The CV curves display all the characteristic regions for catalytically active metals like Pt and Pd: hydrogen adsorption (cathodic scan)—desorption (anodic scan) between –0.3 V and 0 V, the formation of a metal oxides monolayer between 0.5 V and 1.2 V (Pt-O or Pd-O) (anodic scan), and metal oxides reduction peaks (cathodic scan) at 0.37 V for 0.2 mg cm^−2^ Pt, 0.30 V for 0.2 mg cm^−2^ Pd, and 0.29 V for 0.1 mg cm^−2^ Pd + 0.02 mg cm^−2^ Pt. Similar characteristic regions in CV curves appear also for well-dispersed catalysts on carbon supports [39], see Figure 7, where the morphology and structure of such materials are also demonstrated. The shape of the recorded voltammograms is very similar to the ones recorded on carbon substrate, which means that the TiO_2_ nanotubes are well conducting and that there is no barrier for electron transfer at the interface TiO_2_ nanotubes/metal nanoparticles. Any significant resistance R would result in deformed plots because measured potential E would be burdened with the error of ΔE = IR, where I is the current flowing at a given potential. Metal oxides reduction peaks at 0.37, 0.30, 0.29 V were used to estimate the amount of surface atoms of metals deposited onto the catalysts surface. The calculation procedure originates from the paper [38], assuming 2 electrons per metal atom. The electrochemically active surface areas (ECSA) determined with this procedure are given in Table 1. In the last row of the table, data for reference commercial 20% Pd/Vulcan catalyst are shown.

Formic acid electrooxidation of the prepared catalysts was investigated by cyclic voltammetry in 0.5 M HCOOH + 0.5 M H_2_SO_4_ solution, see Figure 6d–f. Note that there are small differences between potential positions of certain features in voltammograms Figure 6a–c and Figure 6d–f because both sets were recorded with different scan rates. There is a marked difference between the behavior of Pd catalyst and that of Pt catalyst towards formic acid electrooxidation: during the anodic scan the reaction starts on Pd already at −0.2 V, while at Pt the current increases only above 0.45 V. Such a large difference in electrochemical behavior on Pd and Pt is a consequence of different reaction mechanisms on both metals. On Pd the electrooxidation follows direct pathway,

HCOOH → CO_2_ + 2H^+^ + 2e^−^

while on the Pt it proceeds through the indirect one

HCOOH → CO_ad_ + H_2_O → CO_2_ + 2H^+^ + 2e^−^

with strongly adsorbed CO_ad_ intermediate blocking the catalyst surface for further reaction [40]. For the potentials between −0.1 V and 0.45 V Pt surface is fully covered with CO_ad_ which renders it completely inactive towards formic acid electrooxidation. The catalyst regenerates at potentials around 0.45 V, where Pt-OH surface groups are formed (see voltammogram Figure 6d) which remove CO_ad_ from the surface oxidizing it to CO_2_. For all the catalysts current passes through maximum, while for the Pt decorated Pd catalyst two separate maxima are observed: the one due to formic acid electrooxidation on Pd at ~0.0 V and the second corresponding to the reaction at Pt at ~0.7 V. Usually, current maxima are observed on a CV plot, because of diffusion limitation, but here it is not the case. Maxima of the formic acid electrooxidation are observed even when the sample in mounted on a rotating disk electrode for which simple theory predicts wave shaped plots [38]. Current decrease with increasing potential takes place because of anions HSO_4_^−^ [41,42] and formate adsorption [43] blocking the surface. At higher potentials 0.7 to 0.95 the metal surfaces are being gradually covered by respectively PdO and PtO single layer, which is also inhibiting the electrooxidation of formic acid. On the reverse scan, the electrodes are inactive until Pt and Pd oxides reduction to metallic Pd and Pt begins (compare voltammograms Figure 6a–c with Figure 6d–f respectively). On the fresh formed metal surfaces, free from adsorbed anions, conditions for the formic acid electrooxidation are most favorable and high maxima are observed on all three samples. For the 0.1 mg cm^−2^ Pd + 0.02 mg cm^−2^ Pt/TiO_2_ NT the specific peak current is as high as 910 mA mg_metal_^−1^. The current decrease during the reverse scan takes place not only because of potential decrease, but also due to readsorption of anions, CO_ad_ on Pt nanocrystals, and surface reconstruction. The detailed shape of voltammograms is the result of all these factors and is therefore difficult to predict. Comparable results and observations were given by the authors of other works, where observed that Pd-Pt nanoparticles exhibited higher formic acid electrooxidation current density during voltametric experiments in comparison to pure Pd [44] and lower start potential of the electrooxidation reactions with reference to pure Pt. Analysis of the data indicates that the addition of Pd enhances the rate of formic acid electrooxidation via a direct reaction mechanism [45].

#### 3.2.2. Fuel Cell Tests

**Figure 8:**
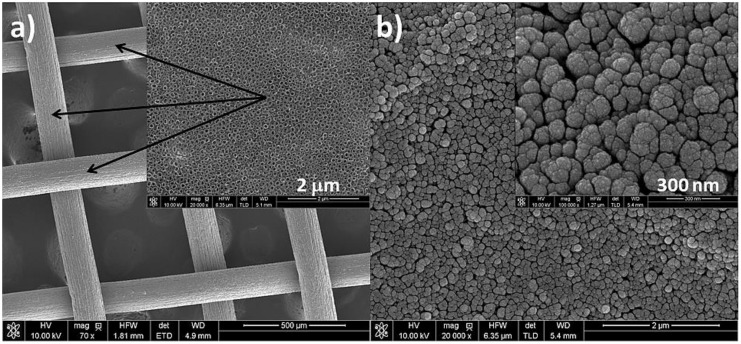
SEM images of a nanotubes formed at 25 V on Ti mesh, annealed at 450 °C/1 h (**a**) and covered with metal deposit: 0.2 mg cm^−2^ Pd (**b**).

The Pd/TiO_2_ nanotubes/Ti mesh catalyst was used as anode for the test reaction in DFAFC fuel cell. This kind of solution allowed the practical use of nanotubes as a carrier for catalytically active metal in the fuel cell. Therefore, the anode was prepared in the same way as Ti foils to produce TiO_2_ nanotubes, but using a platinum cathode in the form of a cylinder in the middle of which was placed a titanium mesh. Pd nanoparticles were subsequently deposited by magnetron sputtering on each side of Ti mesh substrate, as shown in Figure 8. Despite a different starting material—titanium mesh, a similar surface morphology was obtained as for the titanium foil, which is a good reference point to the previously presented electrochemical and materials characterization results.

**Figure 9:**
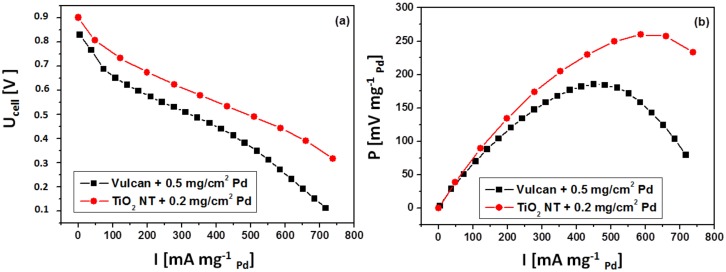
Comparison of the current–voltage curves (**a**) and power vs. specific current (**b**) for electrooxidation of 3 M HCOOH when using Pd/Vulcan or Pd/TiO_2_ as anode catalysts.

Current-voltage curves and power vs. current plots in DFAFC system for Pd/TiO_2_ and Pd/Vulcan (as a reference) anode catalysts are presented in Figure 9. The maximum of specific power for 0.2 Pd/TiO_2_ catalyst is by 70% higher than that of the commercial Pd catalyst. Similar results for the Pd/C catalyst are reported also by Yu and Pickup [31]. This confirms that the nanotube based Pd/TiO_2_ is a promising anode catalyst for DFAFC.

### 3.3. Discussion

The mechanism of electron transfer from Ti support to metal crystallites through TiO_2_ NTs is not evident and so, it deserves a detailed discussion. TiO_2_ is a semiconductor with bandgap of about 3.2 eV (anatase) and, therefore, it should not display any significant electronic conductivity at room temperature. Additionally, the geometry of long NTs with a small cross section seems to be very unfavorable. In spite of that, the TiO_2_ NTs appear to be perfectly conducting. It is well known that the electronic conductivity of TiO_2_ may increase considerably when the material is oxygen deficient or when it absorbs protons. In both cases new states located in the band gap are formed. Such partially reduced TiO_2_ materials are called bronzes and their presence was claimed to be responsible for good performance of Pd/TiO_2_/carbon catalysts [46]. However, in the present case, TiO_2_ NTs were annealed in air and therefore have no oxygen vacancies. As the distance between Ti substrate and the metal particles located on top of the nanotubes is of the order of 1 μm, direct electron tunneling from Ti support to the nanocrystallites located at the top of NTs cannot account for the conductivity observed. However, the STEM photos show numerous metal nanoparticles located along the TiO_2_ nanotubes. Even if they do not form a continuous film on certain TiO_2_ NTs, the electrons can tunnel between the nanoparticles separated by distances of the order of several nanometers. This conclusion is supported by the results of additional experiments, e.g., catalysts with longer TiO_2_ NTs (more than 1.5 μm) were inactive towards formic acid electrooxidation. In 0.5 M H_2_SO_4_, they displayed resistor like CVs without visible Pt and Pd features. In turn, catalysts supported on the TiO_2_ NTs of the same length, but lower Pt loadings (0.02 mg cm^−2^), and so, having less metal nanoparticles on the NTs walls, displayed lower specific activity in FA electrooxidation than the ones presented in this work. Evidently, a certain concentration of metal nanoparticles on TiO_2_ NTs is necessary to ensure efficient electron flow between titanium collector and the metal nanoparticles located mainly on top of TiO_2_ NTs.

It is surprising that the performance of Pd/TiO_2_ NTs catalyst in the fuel cell test is better than that of the reference Pd/Vulcan catalyst which has much smaller and well dispersed Pd nanoparticles (~3 nm) than the Pd/TiO_2_ NTs used in the present work (~10 nm) and, therefore, considerably higher electrochemically available surface (EAS ~60 m^2^ g^−1^ vs. ~10 m^2^ g^−1^).

There are a number of factors which can compensate for such a large surface difference:

(i) One of them could be metal–support interaction which may change electronic properties of metal nanoparticles. Such an interaction should manifest itself by a shift of XPS peak position. However, such evident shift was not observed in the present work. It is instructive to compare the XPS data obtained in this work (no change in the Pt 4f_7/2_ position 71.0 eV) with those from literature [35], where the Pt 4f_7/2_ maximum is shifted to 71.3 eV. The difference results from different Pt deposition methods used in both works. The magnetron sputtering used in the present work produces metal deposits of the thickness of about 150 nm, located mainly on top of TiO_2_ NTs, so the XPS signal comes from surface metal atoms having no direct contact with the support. In turn, metal precursor reduction method used in [35] produces more uniform and so thinner metal deposits and so the XPS signal comes from metal being in contact with the TiO_2_ NTs. Therefore, although such an effect was observed in [35], it cannot account for high specific activity of the TiO_2_ NTs supported catalyst used in the present work.
(ii) It was shown that titanium dioxide is more hydrophilic than the carbon supports commonly used [46], which ensures easier formic acid access to the reaction zone, and facilitates CO_2_ removal. In the case of carbon-supported catalysts, CO_2_ bubbles block the catalyst surface, hindering access of the formic acid [29].
(iii) There is a marked difference in the structures of catalyst layers for the two cases. The standard ink based catalyst layer employed for the carbon supported catalyst is about 10 μm thick and electronic conductivity between carbon support particles separated by larger particles of Nafion ionic conductor does not ensure perfect electron flow between catalyst nanoparticles and the current collector. In contrary, virtually all the Pd aglomerates, located on top of TiO_2_ NTs, have good electronic connection with the Ti collector and ionic (H^+^) contact with Nafion membrane. Also, diffusion of formic acid to the catalyst and removal of the CO_2_ product, are more efficient for a thin layer of metal nanoparticles on top of TiO_2_ NTs, than for much thicker classical catalyst layer with carbon supported catalyst.
(iv) Another factor that works in favor of the TiO_2_ NTs supported catalyst is the difference in crystallite sizes in the two systems. From the paper on crystallite size effect on FA electrooxidation [47] it follows, that the turnover frequency (the reaction rate per 1 surface atom per second) is a complex function of the crystallite size, and, that it is the highest for an optimum crystallite size between 5 and 7 nm. One of the reasons for such a behavior is the fact, that there are two Pd neighboring atoms necessary for dissociative adsorption of FA and, therefore, small carbon supported crystallites (3 nm) having higher contribution of edges are less effective in FA electrooxidation then the larger ones (~10 nm) TiO_2_ NTs supported Pd nanoparticles having higher contribution of crystal planes.

## 4. Conclusions

We have shown that TiO_2_ nanotubular platform obtained by anodization of Ti substrate appears a promising support for metal electrocatalysts. Metal nanoparticles deposited by magnetron sputtering accumulate on the top of the TiO_2_ NTs, forming ‘rings’ composed of nanoparticles agglomerates on the nanotube tops. A part of metal nanoparticles decorates also TiO_2_ NTs walls, thus providing sufficient electronic conductivity for electron transportation between the metal nanoparticle rings and Ti current collector.

CV tests have shown for carbon supported catalysts, that the activity of TiO_2_ NTs decorated with Pd was considerably higher than that one decorated with Pt. For TiO_2_ NTs supported Pd catalyst specific activity (per mg of metal) was higher than that for well dispersed carbon supported commercial catalyst. The tests at DFAFC have revealed that the maximum of specific power for 0.2 Pd/TiO_2_ catalyst was 70% higher than that of the commercial Pd catalyst. This can be ascribed to (1) high hydrophilicity and (2) a suitable morphology of the anodic layer, as well as a closer to optimum Pd particle size resulting in high turnover frequency. As the Ti mesh does not allow to deposit sufficient amount of metal catalyst per geometrical surface unit, further improvement can be made by replacing Ti mesh with a Ti frit or sponge, which will provide macro roughness, in addition to the TiO_2_ NTs forming roughness at the nano and micro scales. Also, changing metal deposition to chemical reduction of a suitable precursor should provide more uniform coverage of the TiO_2_ NTs by the metal catalyst, which should desirably increase the surface available for the electrochemical process of interest.

## Figures and Tables

**Table 1:** Average electrochemically active surface area for samples investigated

Catalyst	Specific Metal Surface Area (m^2^ g_metal_^−1^)
0.2 mg cm^−2^ Pt/TiO_2_ NT	8.5
0.2 mg cm^−2^ Pd/TiO_2_ NT	7.6
0.1 mg cm^−2^ Pd + 0.02 mg cm^−2^ Pt/TiO_2_ NT	11.2
0.24 mg cm^−2^ Pd of commercial Pd/Vulcan	65.1
